# Diuretic and serum electrolyte regulation potential of aqueous methanolic extract of *Solanum surattense* fruit validates its folkloric use in dysuria

**DOI:** 10.1186/s12906-016-1148-3

**Published:** 2016-06-03

**Authors:** Muhammad Masood Ahmed, Shumaila Andleeb, Fatima Saqib, Musaddique Hussain, Most. Nurtaj Khatun, Bashir Ahmad Ch, Hafizur Rahman

**Affiliations:** Faculty of Pharmacy, Bahauddin Zakariya University, Multan, Pakistan; Department of Pharmacology, School of Medicine, Zhejiang University, Hangzhou, China; Department of Zoology, Rajshahi College, National University, Gazipur, Bangladesh; Department of Botany, University of Rajshahi, Rajshahi, 6205 Bangladesh

**Keywords:** *Solanum surattense*, Diuretic activity, Renal canculi, Dysuria

## Abstract

**Background:**

*Solanum surattense* Burm. (Solanaceae) is traditionally used for management of various ailments. The study was conducted for provision of pharmacological justification for folkloric uses of *Solanum surattense* in the treatment of dysuria.

**Methods:**

Rats were randomly divided into 5 groups, each of (*n* = 6). Aqueous methanolic fruit extract of *S. surattense* were also administered intraperitoneally to the rats at doses of 50, 70 and 100 mg/kg. Furosemide (10 mg/kg i.p) was used as standard drug whereas controls were given saline solution (40 mL/kg i.p). The electrolytes in urine were measured using a flame photometer whereas serum sodium, potassium, calcium, bicarbonate and blood urea nitrogen (BUN) were determined by using an automatic analyzer. Urine osmolality was assayed by the micro-osmometer.

**Results:**

The extract *S. surattense* induced diuretic effects in a dose-dependent manner as compared with control. Upon administration of extract (70 and 100 mg/kg), we observed the prominent (*p* < 0.01) increase in the urine volume and osmolality in comparison to control group. However, plant extract (100 mg/kg) significantly increase the urinary electrolyte excretion especially calcium (*p* < 0.05) to that of the furosemide whereas level of magnesium remains constant. Moreover, our results showed a decrease in serum levels of sodium, potassium, calcium and blood urea nitrogen (BUN), but concentration dependent increase in bicarbonate was found in the test groups. There was no substantial change in the pH of urine samples of the extract-treated groups.

**Conclusion:**

These results indicate that *S. surattense* investigated exert its action by causing diuresis in the treatment of dysuria.

## Background

Plants produce a wide range of biologically active molecules which are enriched source of various medicines. In Pakistan and Bangladesh, people use many indigenous herbs and plants as curative drug without any scientific rationale of their beneficial effects. Previously, many studies have been conducted to investigate the pharmacological and phytochemical characteristics of herbs and other plants to provide a scientific approach to their traditional uses [1, 2]. In certain cases, conventional healers use medicinal plant extracts as diuretics. Dearing et al. reported that almost 85 plant species have shown diuretic potential [3]. It has been demonstrated that different crude drugs of plant origin have shown promise to treat various urologic diseases [4]. Dysuria is the condition in which pain is felt during urination due to urinary tract infections [5] and common among people accompanying the findings of renal calculi [6]. Symptomatic treatment of dysuria is primarily based on the use of diuretics, anti inflammatory and antibacterial drugs. In addition, Commission E has granted approval for some plant extracts being used as diuretics [4]. However both in vivo and in vitro studies, the use of traditional therapies as diuretics in different urinary disorders remains to be elusive.

Selection of systematic method for the pharmacological evaluation of botanical products based on their use in the traditional systems of medicine. *Solanum surattense* Burm. Syn. *S. Xanthocarpum* (Solanaceae) locally called as *Kantikari* (yellow berried night shade) is a commonly growing perennial herb and used for curing urologic ailments in both Ayurvedic and Traditional Chinese Medicine system [7–11]. The *S. surattense* fruits are edible and used as diuretic, anti-inflammatory and antiasthmatic [11–13]. The use of root decoction of *S. surattense* as effective diuretic is common among local communities [14, 15]. Later, Siddiqui et al. have found some potential phyto-constituents in *S. surattense* fruits showing the diuretic activities [7]. Moreover, Patel et al. has demonstrated the anti-urolithiatic effects of *S. surattense* fruit extract on rat model of nephrolithiasis as well as having potential to excrete magnesium, calcium oxalate and uric acid [11]. The Ayurveda doctors are extensively using the extract of *S. surattense* fruits and whole plant in their prescriptions to treat dysuria might be due to its diuretic efficiency [7, 9]. The decoction of the entire plant is utilized by local traditional healers to treat gonorrhea in their common practice [9, 10]. The extract of *S. surattense* has shown a strong antibacterial activity against *Pseudomonas aeruginosa* in vitro experimental settings [5, 16]. In previous reports, *S. surattense* treatment showed antioxidant effects by improving the renal function in rat model of urinary lithiasis [11, 15]. Recently, Pandey and his colleagues revealed the scientific reasons for metal phytoextraction potential of *Solanum surattense* plants found in localities near Jharkhand, India [17]*.* The seeds of *S. surattense* are used to treat cough and asthma due to its expectorant action [12] whereas paste of leaves is utilized for reliving pains [13]. *S. surattense* is also used as cardio tonic probably due to alkaloidal glycosides presence [7, 9]. Furthermore, scientific investigations revealed the *S. surattense* fruit shows hypoglycemic effects in normal and diabetic rats [18, 19], wound healing activity [20], physicochemical function [21], cytotoxic activity like various Egyptian plants [22, 23], anthelmintic and antimalarial properties [24]. Phytochemical studies of *S. surattense* berries contain β-sitosterol with phenolic methyl caffeate, caffeic acid and coumarins like aesculetin and aesculin [7]. Other constituents like solasonine, solamargine, solasurine [25], torvoside K and L, khasianine [26], aculeatiside A and solamargine [27] are also found in *S. surattense* fruit.

Previous studies showed that indigenous plants having ethnomedical reputation of diuresis to treat dysuria and hypertension were investigated for their diuretic activity in rats and humans [28–30]. Moreover, the serum electrolytes and blood urea nitrogen alterations have been found upon ‘*Aloe vera’* extract treatment in rats [31]. However, having enough understanding about numerous pharmacological activities of *S. surattense* being used in urinary-lithiasis animal model, but still there is a need to justify the implication of *Kantakari* fruit to induce diuresis and reduce serum electrolytes level.

Although *S. surattense* has traditionally been used to treat dysuria and other urinary ailments, but no pharmacological record is available in order to validate such conventional uses. The current study was done to examine the diuretic potential and probable mode of action which may authenticate the traditional use of the *S. surattense* fruit in dysuria.

## Methods

### Drugs and chemicals

Furesemide, from Sanofi-aventis Pakistan Limited, Karachi, diuretic drug used for reference. Sodium chloride was obtained from BDH Laboratory supplies, Poole, England whereas methanol (70 %) was purchased from Merck (Germany). All other reagents utilized in experiments, were purchased from Sigma Chemicals Co. St Louis, USA. All chemicals were of high purity and grade.

### Plant collection and extraction

The plant of *S. surattense* was collected from Kot Addu, Pakistan, in September 2012 and was identified by taxonomist, Prof. Dr. Altaf Ahmad Dasti of the Institute of Pure and Applied Biology, Bahauddin Zakariya University, Multan (voucher number STW 644). The fruit (berries) of *S. surattense* were separated from plant were washed and dried under shade for 4 weeks. The material was mechanically powdered and passed through a 20-mm sieve after keeping them in an electric oven at 35 °C for 24 h. About 990 g of the grounded plant material was subjected to extraction by triple maceration process with methanol (70 %) in air tight glass jars at room temperature for 10 to 12 days with occasional shaking. The organic waste was separated by percolation with muslin cloth. The obtained liquid was further strained by passing through whatman.no 1 filter paper. This process was repeated for three times to obtain dark brown thick jelly like consistency; named Ss.ME. The moisture was removed by making it in lyophilized form with the help of rotary evaporator (Rotavapor) under reduced pressure at 38 °C to obtain approximate yield of 19.1 %. The extract was kept in light protected air-tight bottle at −4 °C [2].

### Acute toxicity test

Acute toxicity test was performed by using 2, 4, 6, 8 and 10 g/kg of Ss.ME, as previously described [32].

### Preliminary phytochemical screening

Phytochemical evaluation was performed to determine different constituents in extract of *S. surattense* using the methods which are previously described [33, 34]. Mayer’s test was performed and formation of white precipitate (ppt) showed the presence of alkaloids in *S. surattense* extract. Frothing test was accomplished to identify the saponins in extract. Specified tests for tannins and phenols were done. The ppt of green or blue color appeared upon addition of ferric chloride to the extract mixture. Further tests were performed for sterols and tri-terpenoids. The brown ring was appeared at the junction of two layers following addition of few drops of acetic anhydride in initially boiled and then cooled solution of plant extract. The upper layer changed its color to green and red showed the presence of sterols and tri-terpenoids in *S. surattense* extract. [33, 34].

### Animals and housing conditions

Adult albino rats (♂/♀) with average weight 220–240 g were given tap water and diet mixture containing choker, dry milk and chicken feed (locally made) in the ratio of 2:1:2. The rats were placed in polycarbonate cages of size (50 × 35 × 20 cm). The animals were housed under standard temperature (25–28 °C) and humidity conditions. The experiments were performed with the approval of ethical committee of Bahauddin Zakariya University, Multan, Pakistan.

### Dose preparation *S. surattense* extract

The methanolic extract of *S. surattense* was mixed in saline (0.9 %) in a sterilized tube. Then vortex the mixture and kept in Ultrasonic cleaner (Elma, Model LC20H, Germany). Three doses of various concentrations (50, 70 and 100 mg/kg) were made by ultrafiltration and pH determination (Ino Lab, Model pH7310, Germany).

### Measurement of diuretic activity

The method used with minor modification from Gilani et al. [29] was used to determine the diuretic activity. All doses of extract were administered intraperitoneally. Rats were fasted overnight but given access to water. Animals were separated into 5 groups with *n* = 6 in each. The group A (control) was given saline (40 mL/kg), group B, C and D (test group) were given different doses of *S. surattense* extract (50, 70 and 100 mg/kg) while group E was treated with furosemide (10 mg/kg) used as reference. After treatment, the animals were transferred in individually ventilated separate cages (Techniplast, Italy). The graduated vials were used for urine collection whereas volume was measured at 1, 2, 4 and 6 h and monitored hourly. The mean urine volume was calculated and expressed as mL/100 g/body weight [29, 35].

### Urinary electrolyte and osmolality analysis

Sodium, potassium, calcium, magnesium excretion level and pH were measured in urine sample of rat at the end of the mentioned experimental time. The concentration of sodium and potassium was expressed in mol whereas μmol and mmol/100 g body weight for calcium and magnesium. Electrolyte levels were determined by using a standardized flame photometer (Corning, Model 410 Classic, UK) [29, 35]. The pH of urine samples was noted with pH meter [36]. Urine osmolality was analyzed by micro-osmometer (Model 3350, Advanced Instruments, Inc.) with minor alternation in method [29].

### Blood sample collection

The blood samples from the animals were initially obtained through the retro-orbital plexus, after which they were killed by cervical dislocation and more blood was collected via heart puncture. The blood sample collected from each rat was put in lithium-heparinized sample bottles. Serum was centrifuged at 3000 rpm at 4 °C for 10–15 min [31].

### Determination of serum electrolytes, bicarbonate and blood urea nitrogen (BUN)

Serum sodium, potassium, calcium, bicarbonate and blood urea nitrogen were measured by an automatic analyzer (MSLAB08, MSL, China).

### Statistical analysis

The results are expressed as mean ± S.E.M (Standard error mean). The significance between the test groups and the control group were analyzed using unpaired *t*-tests. Data were analyzed using Graph Pad Prism. The results were taken significant statistically when *p* < 0.05.

## Results

### Phytochemical evaluation of *S. surattense*

Aqueous methanolic extract of *S. surattense* was analyzed to check different pharmacologically active phytoconstituents and we found that the extract contains alkaloids, tannins, saponin, sterols and tri-terpenoids whereas anthraquinones were absent.

### Acute toxicity test

Acute toxicity of S*. surattense* fruit extract was checked at various doses (2, 4, 6, 8 and 10 g/kg). We found no change in animal behavior and mortality up to the maximum dose i-e 10 g/kg. These observations indicated that the extract was safe up to the highest test dose.

### Urine volume and pH

The methanolic extract of *S. surattense* (100 mg/kg) and standard furosemide showed significant diuretic activity versus control at the 6^th^ h (*p* < 0.01; 2.72 ± 0.09 mL) and (*p* < 0.01; 3.54 ± 0.13 mL), respectively. The lowest test dose (50 mg/kg) of Ss.ME also showed marked diuretic activity (*p* < 0.05; 2.08 ± 0.12 mL) versus control at the 6th h. The urine output was also significant (*p* < 0.05) during the 2^nd^, 4^th^ and 6^th^ h after treatment with the dose (70 mg/kg) of Ss.ME (Table 1). The pH of urine samples after administration of Ss.ME 50, 70 and 100 mg/kg doses was 6.5, 6.8 and 6.7, respectively. We found no significant change in mean urinary pH.

**Table 1:** Effect of *S. surattense* fruit extract on urine volume

Treatment	Urine volume (ml/100 g of body weight)			
	1 h	2 h	4 h	6 h
Saline 40 mL/kg	0.74 ± 0.32	0.96 ± 0.17	0.64 ± 0.13	1.16 ± 0.15
*S. surattense* 50 mg/kg	0.85 ± 0.15	1.12 ± 0.13	1.30 ± 0.17	2.08 ± 0.12*
*S. surattense* 70 mg/kg	1.08 ± 0.14	1.23 ± 0.18	1.62 ± 0.15*	2.34 ± 0.16*
*S. surattense* 100 mg/kg	1.26 ± 0.11	1.52 ± 0.14*	1.86 ± 0.13*	2.72 ± 0.09**
Furosemide 10 mg/kg	1.94 ± 0.17	2.34 ± 0.15*	2.93 ± 0.10*	3.54 ± 0.13**

Each value is expressed as a mean ± S.E.M (*n* = 6) *p < 0.05 and **p < 0.01

### Osmolality of urine

As shown in Table 2, the osmolality of urine of the control group remained constant, irrespective of time; however the animals administered with Ss.ME showed significant rise in osmolality of urine than that of the controls. With an exception of doses 70 and 100 mg/kg induce a significant urinary osmolality (*p* < 0.05) during the second hour. However, the test dose of Ss.ME (70 mg/kg) showed highest osmolality (*p* < 0.01; 452 ± 25 mOsm) at 6^th^ h. Moreover, the furosemide-treated group showed a significant rise in the osmolality of urine time dependently, with a maximum increase during the 4^th^ h (*p* < 0.01; 645 ± 36 mOsm).

**Table 2:** Effect of *S. surattense* fruit extract on Osmolarity

Treatment	Urine osmolality (mOsm)			
	1 h	2 h	4 h	6 h
Saline 40 mL/kg	162 ± 28	194 ± 36	203 ± 33	282 ± 68
*S. surattense* 50 mg/kg	175 ± 45	245 + 28	285 ± 35*	328 ± 57*
*S. surattense* 70 mg/kg	210 ± 32	306 ± 35*	294 ± 26	452 ± 25**
*S. surattense* 100mg/kg	260 ± 55	421 ± 42*	365 ± 38*	372 ± 30
Furosemide 10 mg/kg	320 ± 70	380 ± 85*	645 ± 36**	390 ± 58

Each value is expressed as a mean ± S.E.M (*n* = 6) *p < 0.05 and **p < 0.01

### Electrolytes excretion level in urine

The urinary sodium and potassium level was measured at 1, 2, 4 and 6 h whereas calcium and magnesium concentration was analyzed after 6^th^ h from the urine samples, as shown in Tables 3 and 4. Overall, the urine content of these electrolytes of the control animals was same. Among the three doses of extract tested, significantly higher (*p* < 0.01) concentration of the urinary Na^+^ (291 ± 23 mol) and K^+^ (79 ± 7.5 mol) excretion was observed in the animals treated with dose 100 mg/kg of Ss.ME at the 6^th^ h. However, the significant (*p* < 0.05) amount of Na^+^ and K^+^ excreted in the urine after treatment with Ss.ME during the 2^nd^ and 4^th^ h. Furosemide caused significant increase in Na^+^ and K^+^ excretion in urine during every hour control group whereas a marked increment (*p* < 0.01) in the urinary Na^+^ (305 ± 27; 324 ± 15 mol) and K^+^ (89 ± 13; 91 ± 20 mol) levels were noted after furosemide treatment at the 4^th^ and 6^th^ h, respectively (Table 3).

**Table 3:** Total excretion of sodium and potassium in urine over a period of 6 h after treatment with *S. surattense* fruit extract

Treatment	Na^+^(mol)				K^+^ (mol)			
	1 h	2 h	4 h	6 h	1 h	2 h	4 h	6 h
Saline 40 mL/kg	62 ± 38	114 ± 25	106 ± 10	98 ± 29	21 ± 5	35 ± 7	28 ± 10	33 ± 11
*S. surattense* 50mg/kg	88 ± 13	107 ± 17	127 ± 25	132 ± 16*	24 ± 7	38 ± 6	59 ± 11*	65 ± 10*
*S. surattense* 70mg/kg	111 ± 9	128 ± 16	181 ± 19*	257 ± 20*	33 ± 6.5	47 ± 13	63 ± 16*	69 ± 14*
*S. surattense* 100mg/kg	120 ± 21	184 ± 14*	192 ± 32*	291 ± 23**	42 ± 8	58 ± 15*	68 ± 8.5*	79 ± 7.5**
Furosemide 10 mg/kg	144 ± 35	247 ± 26*	305 ± 27**	324 ± 15**	66 ± 5.9*	78 ± 9*	89 ± 13**	91 ± 20**

Each value is expressed as a mean ± S.E.M (*n* = 6) *p < 0.05 and **p < 0.01

**Table 4:** Effect of *S. surattense* fruit extract on urinary calcium and magnesium

Treatment	Ca^2+^ μmol/6 h	Mg^2+^ mmol/6 h
Saline 40 mL/kg	6.68 ± 4.52	0.08 ± 0.02
*S. surattense* 50 mg/kg	12.32 ± 5.14*	0.07 ± 0.01
*S. surattense* 70 mg/kg	18.17 ± 3.91*	0.07 ± 0.01
*S. surattense* 100 mg/kg	29.16 ± 2.95**	0.08 ± 0.02
Furosemide 10 mg/kg	36.20 ± 5.75**	0.09 ± 0.01

Each value is expressed as a mean ± S.E.M (*n* = 6)*p < 0.05 and **p < 0.01

At the 6^th^ h, urinary calcium (Ca^2+^) excretion was found to be significantly higher in the Ss.ME and furosemide treated groups. However, the highest calcium excretion level in urine after treatment with Ss.ME dose 100 mg/kg (*p* < 0.01; 29.16 ± 2.95 μmol) and furosemide treated group (*p* < 0.01; 36.20 ± 5.75 μmol) was noted than that in the control group. Moreover, no significant difference was observed among control, test and furosemide treated groups regarding the 6^th^ h urinary excretion of magnesium (Mg^2+^) content (Table 4).

### Serum level of sodium, potassium, calcium, bicarbonate and blood urea nitrogen (BUN)

**Fig. 1:**
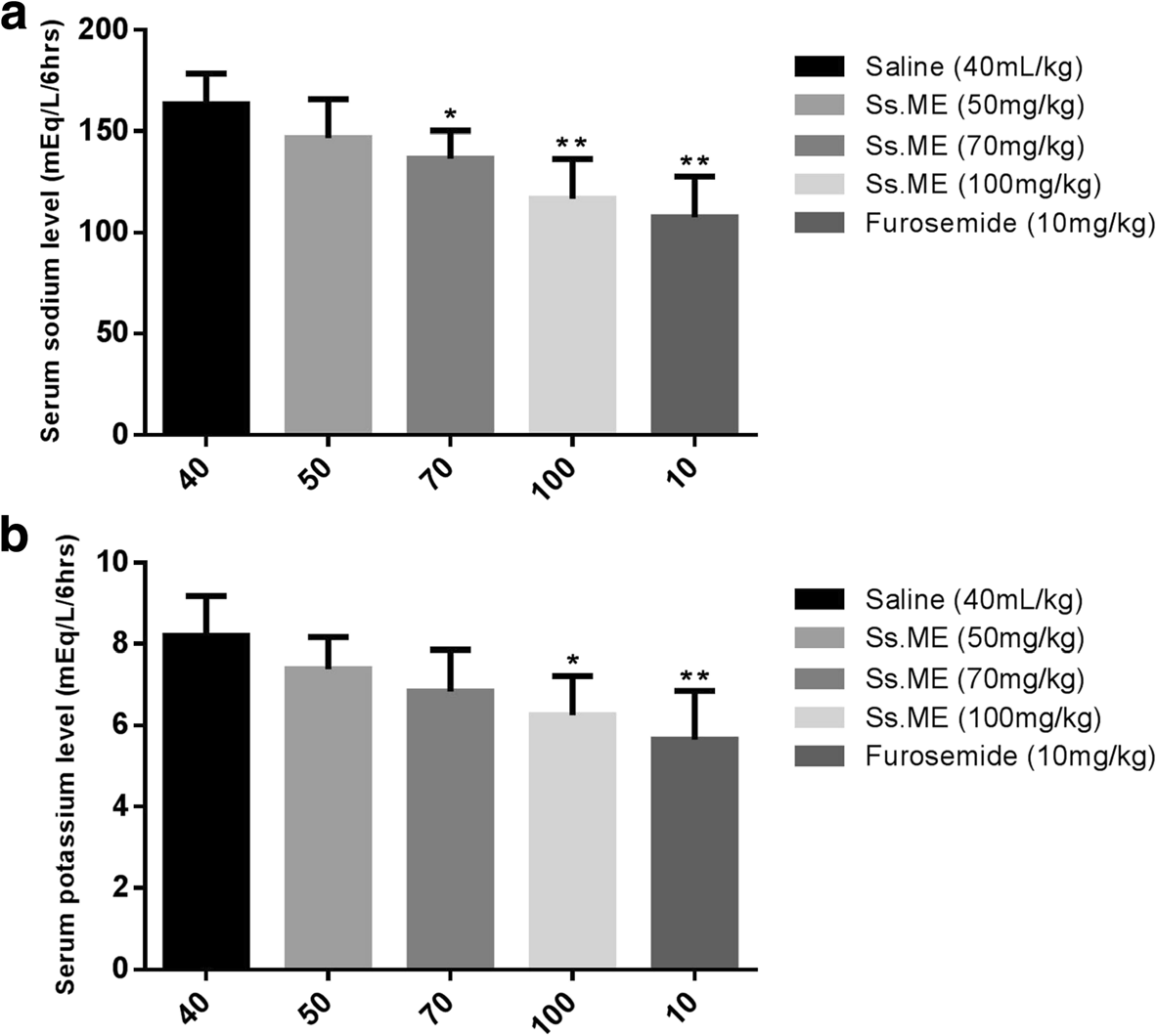
Effect of *Solanum surattense* fruit extract (Ss.ME) and furosemide on serum levels of **a** Na^+^ and **b** K^+^ in rats. Values are shown as mean ± S.E.M., *n* = 6. *p < 0.05 and **p < 0.01 versus control

**Fig. 2:**
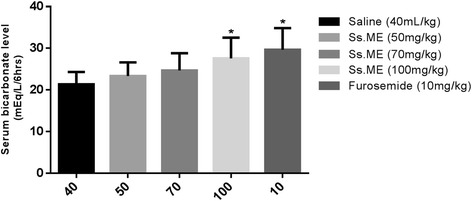
Effect of *Solanum surattense* fruit extract (Ss.ME) and furosemide on serum levels of bicarbonate in rats. Values are shown as mean ± S.E.M., *n* = 6. *p < 0.05 and **p < 0.01 versus control

**Fig. 3:**
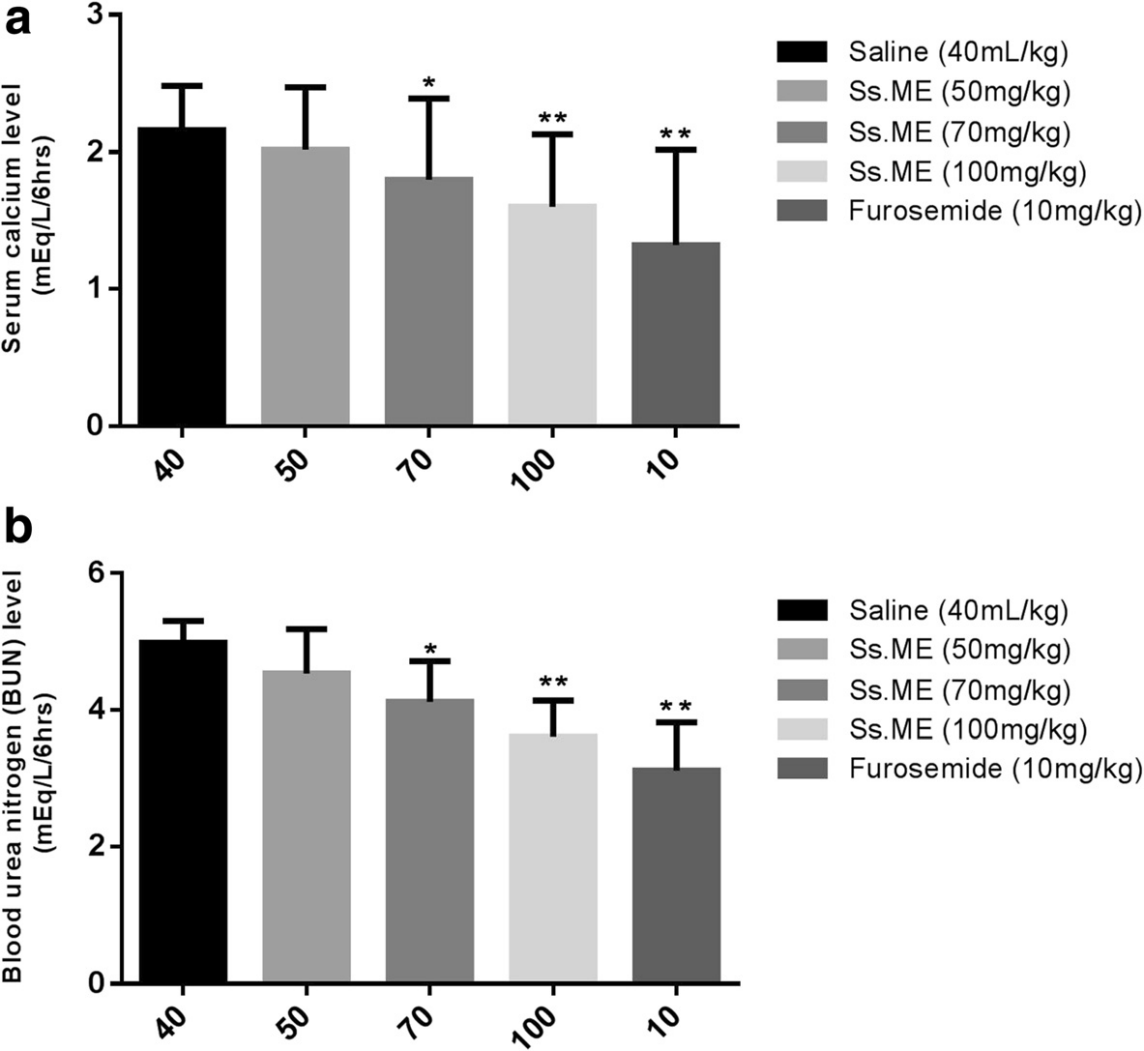
Effect of *Solanum surattense* fruit extract (Ss.ME) and furosemide on **a** serum Ca^+2^ and **b** blood urea nitrogen (BUN) in rats. Values are shown as mean ± S.E.M., *n* =6. *p < 0.05 and **p < 0.01 versus control

Among three test groups, we observed concentration dependent decrease in the serum levels of sodium (*p* < 0.01) and potassium (*p* < 0.05) in the test group after treatment with Ss.ME dose 100 mg/kg and furosemide treated group (*p* < 0.01) as compared to the controls (Fig. 1a and b). We found significant increase in serum bicarbonate (*p* < 0.05) in 100 mg/kg Ss.ME and furosemide treated animals in comparison to control group (Fig. 2). However, there was marked decline in the serum calcium and blood urea nitrogen level (*p* < 0.01) in the rats after administration of Ss.ME dose 100 mg/kg and furosemide comparable to test dose 70 mg/kg (*p* < 0.05) than that of control group (Fig. 3a and b).

## Discussion

The present study was under taken to validate the folkloric use of *S. surattense* fruit in dysuria by evaluating its diuretic activity.

The results of our study validated that *S. surattense* aqueous methanolic extract exhibited a significant but dose dependent rise in the urine output in rats, like standard diuretic drug, furosemide [36]. Similarly, with increase in the urine volume, Ss.ME also boosted the excretion of Na^+^ K^+^ and Ca^2+^ in urine, as triggered by furosemide when compared with controls. Loop diuretics not only increase the urinary outflow, are also known to increase the electrolyte excretion in urine, called as saluretic [36] whereas the presence of diuretic constituents are likely to supplement the use of *S. surattense* in dysuria [7, 11]. The active constituent responsible for diuretic activity of the extract has not yet been found, but the phytochemical assay revealed that *S. surattense* contains alkaloids, phenols, saponins and sterols [7, 37]. The chemical structures and pharmacological properties of tannins and saponins are so intricate. However, the diuretic activity of the plant extract might be due to initiation of multiple mechanisms. Previous studies demonstrated that tannins in plants have shown both diuretic and vasodilator potential [31]. Previously, it has been reported that root extract of *Ananas comosus* enhanced diuresis and osmolality which was not dependent on salt content in plant [28]. Consistent to the previous findings, the different doses of Ss.ME increases the osmolality of urine together with excretion of electrolytes per unit time may help to investigate the mechanisms by which *S. surattense* shows diuretic action [11].

Furthermore, we investigated the various concentrations of Ss.ME on renal function by analyzing the serum level of electrolytes and blood urea nitrogen. However, recent studies showed that *S. surattense* fruit extract acted as anti-urolithiatic in rats [11]. The result from this study demonstrated that Ss.ME treatment induced a change in electrolyte levels in experimental animals as explained in previous report [31]. Moreover, it has been proved that electrolytes play an important role in muscle contraction and relaxation [38]. Saka and colleagues has shown the reduction in sodium and potassium ions is correlated with the increased bicarbonate level after plant extract treatment [31]. Here, we also found Ss.ME dose dependent significant reductions in the serum level of sodium, potassium and calcium whereas serum bicarbonate concentration is increased. whereas These results show that Ss.ME induce alterations in electrolytes level evident by low plasma level of sodium, and rise in bicarbonate level though significant, was not sufficient to cause metabolic alkalosis [31]. Previous studies showed that high protein and fat diets play a key role in the pathogenesis of calcium-containing renal stones and increases blood urea nitrogen [5, 11]. The plasma concentrations of urea and calcium could be used as indicator of renal function. However, we found the substantial decreases in blood urea nitrogen (BUN) and calcium after *S. surattense* fruit extract treatment whereas similar parameters have been found in rats with urinary-lithiasis [11]. The decline in serum calcium and urea concentrations depicts that Ss.ME dose dependently enhances the renal function.

It has been documented that saponins showed diuretic effect in connection with steroids and vitamin D while tannins have shown the diuretic and vasodilator effects [39]. Therefore, we can suggest that the diuretic potential of the methanolic extract of *S. surattense* is due to the presence of phenolic caffeic acid and methyl caffeate as secondary metabolites [7, 11]. However, antibacterial effect of *S. surattense* plant extract against *Pseudomonas aeruginosa* [5, 16] most probably linked with diuretic action of the Ss.ME to treat dysuria [11]. Hence, further studies are required to elucidate the efficacy and efficiency of *Solanum surattense* berries.

## Conclusion

By the findings of our study, we can conclude that *S. surattense* exhibits substantial diuresis when compared to that of furosemide whereas decline in serum calcium and blood urea nitrogen levels provide strong mechanistic background for its diuretic potential in dysuria treatment.

## Abbreviations

BUN, blood urea nitrogen; Ca^2+^, calcium; K^+^, potassium; i.p, intraperitoneally; Mg^2+^, magnesium; ml, milliliter; mOsm, milliosmole; mmol, millimole; Na^+^, sodium; ppt, precipitate Ss.ME, methanolic extract of *Solanum surattense*; S.E.M, standard error mean; μmol, micromole
